# Practical recommendations for implementing a Bayesian adaptive phase I design during a pandemic

**DOI:** 10.1186/s12874-022-01512-0

**Published:** 2022-01-20

**Authors:** Sean Ewings, Geoff Saunders, Thomas Jaki, Pavel Mozgunov

**Affiliations:** 1 Southampton Clinical Trials Unit, University of Southampton, Mailpoint 131, Southampton General Hospital, Tremona Road, Southampton, SO16 UK; 2 MRC Biostatistics Unit, University of Cambridge, Cambridge, UK; 3 Department of Mathematics and Statistics, Lancaster University, University of Lancaster, Lancaster, UK

**Keywords:** Bayesian, Phase I, Adaptive design, Dose escalation

## Abstract

**Background:**

Modern designs for dose-finding studies (e.g., model-based designs such as continual reassessment method) have been shown to substantially improve the ability to determine a suitable dose for efficacy testing when compared to traditional designs such as the 3 + 3 design. However, implementing such designs requires time and specialist knowledge.

**Methods:**

We present a practical approach to developing a model-based design to help support uptake of these methods; in particular, we lay out how to derive the necessary parameters and who should input, and when, to these decisions. Designing a model-based, dose-finding trial is demonstrated using a treatment within the AGILE platform trial, a phase I/II adaptive design for novel COVID-19 treatments.

**Results:**

We present discussion of the practical delivery of AGILE, covering what information was found to support principled decision making by the Safety Review Committee, and what could be contained within a statistical analysis plan. We also discuss additional challenges we encountered in the study and discuss more generally what (unplanned) adaptations may be acceptable (or not) in studies using model-based designs.

**Conclusions:**

This example demonstrates both how to design and deliver an adaptive dose-finding trial in order to support uptake of these methods.

**Supplementary Information:**

The online version contains supplementary material available at 10.1186/s12874-022-01512-0.

## Background

Identification of the correct dose of a treatment is an essential part of drug development. A rich literature [1–3] exists highlighting the benefits of model-based [4–6], model-assistant [7, 8] and curve-free approaches [9, 10] over traditional, simple rule-based methods (such as the 3 + 3 design) for recommending a dose for efficacy testing. However, the uptake of these new methods has been slow [11] and several obstacles to their use have been identified, including software, knowledge, and implementation [12–14]. To address some of these challenges, several software solutions have been described and made available [15, 16] and publications describing these methods [17] or experiences using them [18, 19] have been published in recent years.

## Methods

The aim of this paper is to provide further insight into the implementation of model-based designs, and guidance on decisions made throughout the design and analysis of trials using these methods. This includes the specific decision-making for an example trial, as well as guidance that would apply to other trials using similar designs.

The setting for this paper is the AGILE platform trial [20], which seeks to find a safe dose range and preliminary efficacy testing for a number of potential treatments for COVID-19; in particular, we focus on one of the treatments (Molnupiravir) undergoing testing in AGILE. The AGILE trial team includes experienced statistical methodologists and the statistics team within a UKCRC Clinical Trials Unit, who collaborated to help contribute to the design and delivery of the AGILE platform trial. We believe this example highlights that, despite the challenging circumstances of an ongoing pandemic, model-based dose-finding designs can be implemented even when previous experience with such approaches is limited. Additionally, we seek to highlight the added benefit of the model-based approach in terms of decision making, even when set against the steep initial learning curve and the additional resource initially required. At the same time our example shows that pairing with an experienced team is useful to expedite the learning required.

### AGILE platform trial

The AGILE trial is a randomised seamless phase I/II trial platform in which multiple different candidate treatments for COVID-19 can be evaluated [21] (clinicaltrials.gov registration number NCT04746183; registered 09/02/2021). Each of the novel treatments entering the platform undergo a dose-finding phase evaluating a range of safe doses (Phase I) to recommend a dose for further testing, and a group-sequential phase establishing the activity of a dose (Phase II). Below, we describe the statistical methodology used to govern dose-escalation decisions in Phase I for treatments entering the platform (as described in the AGILE master protocol) and describe how it was shaped to fit the setting of one particular treatments, Molnupiravir. We also present some results from the trial for demonstration purposes.

### General dose-escalation setting in AGILE

Based on an increasing body of evidence for their efficient use of information in Phase I trials [22], a model-based dose-finding design was chosen for AGILE. The standard approach involves modelling the dose-toxicity relationship for *m* increasing doses, *d*_*1*_*,d*_*2,*_*,...,d*_*m*_ of a treatment. One major difference in AGILE was that knowledge of COVID-19 symptomatology was still in its infancy during the design of the study, and the rates and types of symptoms could not reliably be separated from treatment side effects (dose-limiting toxicities; DLTs). This motivated the use of a control arm, to avoid labelling potential treatments as unsafe due to misclassifying non-treatment related symptoms [23]. The primary goal of the dose-escalation was therefore to evaluate the additional risk of a dose limiting toxicity (ARDLT), defined in terms of the expected difference in DLT risk between the treatment doses and the control (which constituted standard of care for Molnupiravir). Participants were therefore randomised to control or to the current dose being evaluated to provide robust estimates of ARDLT compared to control. However, many of the considerations we present through this example will also apply to a non-randomised setting.

### Model-based dose-escalation design in AGILE

To model the dose-toxicity relationship, a randomised Bayesian model-based dose-finding design [24] was used. The basic model used to estimate the risk of toxicity, *p*_*j*_, for dose *d*_*j*_ was a two-parameter logistic model. This model assumes that the risk of toxicity increases monotonically with dose. The primary motivation for using this model over the one-parameter model [25] is based on its flexibility. In the randomised setting, the majority of the data during the escalation will be collected around two points on the dose-toxicity curve: around the control DLT rate and in the neighbourhood of the estimated target dose. While the one-parameter model has been found to be flexible enough to accurately approximate the dose-toxicity relationship in the neighbourhood of one point [26] (i.e., in a non-randomised dose-escalation setting), this may not translate to data collected around two points. An extensive simulation study comparing several parametric curves for the dose-toxicity relationship in the randomised setting found that the one-parameter model tended to identify significant differences between the target dose and the control, particularly in scenarios with no or little difference in toxicity (i.e., flat dose-toxicity curve) [24].

The two-parameter logistic model of the risk of toxicity, *p*_*j*_, at dose *d*_*j*_ is given by:

$${p}_j\left({d}_j,{\theta}_1,{\theta}_2\right)=\frac{\exp \left({\theta}_1+{\theta}_2\ast {d}_j\right)}{1+\exp \left({\theta}_1+{\theta}_2\ast {d}_j\ \right)}, for\ j=0,1,\dots, m,$$

where *θ* = (*θ*_1_, *θ*_2_) are the parameters of the model and *d*_*j*_ are the standardised dose levels. The standardised dose levels are found by solving the above equation based on prior estimates of *θ* and *p*_*j*_, (see “Model evaluation”) to ensure that the prior estimates of DLT risks and corresponding dose levels can be aligned with the chosen model shape. Once calculated prior to the trial starting, the levels are subsequently used in the calculations throughout the trial as the parameters, *θ* (and hence the estimated *p*_*j*_), are updated once data is collected. The standardised dose level for the control (*d*_0_) was chosen to be 0, ensuring that the estimate of the dose-toxicity relationship does not affect the DLT risk estimate on standard of care [24]. This means *θ*_1_ can be interpreted as the risk of DLT in the control arm (on the logit scale; i.e., *logit*(*p*_0_) = *θ*_1_), and *θ*_2_ is the steepness of the dose-toxicity curve on the treatment arm. The advantage of including the control arm in the modelling is that it allows for borrowing of information in the estimation of ARDLTs for the non-zero doses. The standardised dose levels were constructed for each treatment in the AGILE platform separately.

The model parameters are estimated using Bayes theorem, using prior distributions (specified by statisticians in communication with clinicians – see “Application of design to Molnupiravir”) on *θ*_1_ and *θ*_2_. We refer the reader elsewhere for more technical details of the estimation for this model [23]. Using the specified dose-toxicity model, dose escalation proceeds as follows:

1. A cohort of *n* participants are randomised between the starting dose (in this case, *d*_*1*_) and control (of sizes *n*_*1*_ and *n*_*2*_, respectively), and DLT outcomes are evaluated.
2. Given observed DLTs, the distributions of the model parameters are updated, and the posterior distribution of the toxicity risk, *p*_*j*_, is obtained for each dose level (including *d*_*0*_).
3. The set of safe doses is found. A dose is deemed to be safe if the ARDLT has a sufficiently low probability of being unacceptably high, i.e.,

$$P\left({p}_j-{p}_0\ge {\gamma}_{toxic}\right)<{c}_{overdose}$$

where *p*_*j*_ − *p*_0_ represents the ARDLT of dose *d*_*j*_ over *d*_*0*_, *γ*_*toxic*_ is the highest ARDLT that is considered to be acceptable in the patient population, and *c*_*overdose*_ is the probability threshold that controls the overdose and defines the stringency of the safety constraints.

4. Subsequent cohorts of patients are assigned to the dose level with highest probability of lying within a pre-defined interval centred around a target ARDLT (*γ*), given by

$$P\left({p}_j-{p}_0\in \left[\gamma -\delta, \gamma +\delta \right]\right)$$

where *δ* is the half-width of the interval (provided that this dose meets the above safety criterion).

5. Steps 2–4 are repeated until the maximum number of patients, *N,* is reached or all doses are deemed unsafe

The choices of the parameters *n*_1,_*n*_2_, *γ*, *γ*_*toxic*_, *δ*, *c*_*overdose*_, together with the prior estimates of toxicity and prior distribution of model parameters, are defined for each treatment entering the AGILE platform, prior to the start of the treatment-specific trial. These choices depend on many factors, e.g., number of doses, dosages, patient populations, and mechanism of action of the compound. Below, we describe how this generic design setting was shaped for Molnupiravir to guide the dose-escalation decisions.

### Application of design to Molnupiravir

To apply the design described above to the given drug, a number of parameter choices must be made. These choices may either be fixed in advance or evaluated via simulation and calibration, designed to achieve desirable model performance (see “Parameter choices based on calibration” onwards). We approached parameter choice in a particular order, based firstly on parameters relating to safety considerations (*γ*, *γ*_*toxic*_, *δ*), then existing knowledge of the treatment (number of doses, *m*), practicalities of the trial (cohort and overall sample size), and finally statistical considerations (parameters of the prior distributions). The prior estimates of DLT risks at each dose (the dose-toxicity skeleton) may be chosen based on existing knowledge of the treatment or treated as a flexible set of parameters chosen based on solely statistical considerations (or some hybrid); as we explain later, we determined the skeleton based on statistical considerations.

The parameters relating to safety require clinical input, and so the discussion of these, in our opinion, should supersede the decision on any other design parameters. These should be kept fixed throughout the trial.

Practical considerations, such as cohort and/or overall sample size, can be evaluated via simulation over a range of values agreed with the research team to understand the trade-off between model performance and recruitment demands. Cohort sizes would typically be small (similar to A + B designs) to allow for frequent reviews of safety and dose-escalation decisions. Parameters relating to statistical considerations can be calibrated via simulations, where a range of values for each parameter are evaluated collectively to determine the set that provides best model performance.

We discuss the details of how we determined the parameters for the Molnupiravir trial in the following sections, with a summary given in Table 1. Note that while we describe our process in a randomised setting, these or similar parameters will need to be specified for non-randomised dose-escalation trials, with the underlying considerations being the same.

**Table 1:** Summary of parameters to be determined, and methods and considerations for the choices made for Molnupiravir

Parameters	Motivation and method for choosing	Considerations	Chosen Value
Target (additional) toxicity rate, *γ*	Motivated by safety considerations. Clinical decision	The DLT definition should be consciously and extensively specified, with acceptable toxicity determined by the clinical context (e.g., availability of other treatments, severity of disease)	20%
Tolerance around target (additional) toxicity rate, *δ*	Motivated by safety considerations. Clinical decision	Any given set of doses is unlikely to contain a dose with DLT rate exactly equal to the target, so flexibility around the target should be considered. As above, this should consider the clinical context	5%
Upper (additional) toxicity bound, *γ*_*toxic*_	Motivated by safety considerations. Clinical decision	Level of unacceptable toxicity (here, toxicity above the control)	30%
Number of Doses, *m*	Motivated by knowledge of treatment. Clinical decision	Number of doses should ensure that the dose-toxicity curve is adequately explored	4 (300 mg bd, 400 mg bd, 600 mg bd, 800 mg bd)
Cohort size	Safety and practical considerations. Options can be evaluated using simulations based on discussion with clinicians	The number of participants the clinicians are comfortable dosing between decisions on dose escalation; how often the model will be updated. Results for various cohort sizes can be shown to clinicians	6 (4 on treatment, 2 control)
Sample Size, N	Practical considerations. Options can be evaluated using simulations based on discussion with clinicians	Sample size should ensure an accurate selection of target doses with high probability	30
Threshold controlling overdosing, *c*_*overdose*_	Simulations; reference in the literature	The value from the literature for the 2-parameter logistic dose-toxicity model was chosen (to speed up simulations)	25%
Hyperparameter *μ*_1_ – the mean of the prior distribution for *θ*_1_	Historical information	Modelled as random variable to account for the uncertainty early in the pandemic	*μ*_1_ = *logit*(0.1) (fixed by 10% DLT rate on control)
Hyperparameters *μ*_2_, *σ*_1_, *σ*_2_ – mean of prior for *θ*_2_ and standard deviations of priors for *θ*_1_ and *θ*_2_	Simulations	Calibrated over a set of feasible dose-toxicity scenarios	*μ*_2_ = − 0.05, *σ*_1_ = 1.10, *σ*_2_ = 0.30
Prior estimates of DLT risk on each dose (also known as the dose-toxicity skeleton), ${p}_j^{(0)}$	May solely reflect existing knowledge of treatment doses, or may be evaluated via simulations	If determined by simulation, the DLT risks on each dose should still align with existing knowledge	17.5, 25, 32.5, 40%

### Parameter choices based on trial setting

The first set of parameters (*γ*, *δ* and *γ*_*toxic*_) should be defined by clinical members of the research team. The target ARDLT (*γ*) was defined after extensive discussions with clinicians, where it was agreed that the target ARDLT should be *γ* = 20%. Furthermore, it was concluded that any doses with additional risk between 15 and 25% would be a reasonable target (as there is no guarantee the doses being evaluated would have an ARDLT of 20%). This provides a half-width of the interval, *δ*, of 5%. It was also agreed by the clinicians that an ARDLT of over 30% would not be acceptable for the studied patient population. Hence, *γ*_*toxic*_ = 30% was chosen. These values were based on the clinical context of potentially severe illness and a lack of alternative treatment options.

Next, the doses to be studied in the trial were defined, based on existing knowledge of the drug. This again requires clinical input and is required before an efficient design (from a statistical point of view) can be determined. For Molnupiravir, doses of 300 mg bd, 400 mg bd, 600 mg bd, and 800 mg bd (with corresponding standardised dose levels denoted by *d*_*1*_*, d*_*2*_*, d*_*3*_ and *d*_*4*_, respectively) were chosen for evaluation. When considering dose escalation, it was agreed that doses could be skipped but cannot be more than doubled, meaning that, if deemed acceptable by the model and by the clinicians, escalation from *d*_*1*_ to *d*_*3*_ and from *d*_*2*_ to *d*_*4*_ were possible. We emphasise that any dose-skipping constraints be defined at this point of the planning, before any numerical evaluations, as it might affect the performance of the design noticeably, especially with limited sample sizes.

Having defined the safety and treatment parameters, we next determined the cohort sizes for a given dose (i.e., the number of patients to be randomised between treatment and control for a given dose). While the cohort size should normally be one of the parameters of the design, assessed by simulations of the operating characteristics under various cohort sizes, this evaluation was primarily done as part of the general AGILE platform design [23] rather than for Molnupiravir specifically. (Note, operating characteristics refers to different criteria to judge model performance, e.g., the ability to select the correct dose under different scenarios – see “Plausible dose-toxicity scenarios”) Specifically, the cohort size of 6 patients with 2:1 randomisation ratio in favour of the experimental arm was found to result in good operating characteristics due to a more balanced (compared to 1:1 ratio) allocation of patients overall between treatment and control (as each cohort contributes patients to the control arm). Importantly, beyond the statistical considerations (i.e., good operating characteristics), it was agreed with the clinicians that they would be happy to make dose-escalation decisions based on this cohort size.

Following this, we next determined the maximum sample size, *N,* of patients that could be enrolled in the study. To ensure desirable operating characteristics, a range of *N* should be evaluated via extensive simulations. In the general AGILE platform evaluations [23], it was found that *N* equal to the number of doses in the study (4 for Molnupiravir) plus 1, multiplied by the cohort size (6; for *N* = 30), resulted in good operating characteristics across various scenarios. Intuitively, this is the minimum sample size that would be enough to reach the highest dose (if deemed safe to escalate to it) while accounting for the fact that a DLT can be observed even on safe doses. This value was used for the preliminary evaluation but due to the platform nature of the trial, no cap was formally specified in the protocol to allow for more flexibility.

Next, the threshold controlling overdosing *c*_*overdose*_ should be selected. Again, while this can be treated as a parameter to be varied (where a value providing desirable balance between accuracy and number of patients exposed to toxic doses can be found via simulations), we fixed this value at *c*_*overdose*_ = 0.25 as it was previously found [27] that this value, applied within the two-parameter logistic model, allows for safeguarding of patients. Subsequent extensive simulation study for the AGILE platform has also confirmed that this value limits the exposure of patients to overly toxic doses [23]. The decision to fix this value was also a pragmatic one, as it reduced the number of parameters to be evaluated and thus reduced the computational complexity and time to run simulations (a particular concern given the context of the trial in an ongoing pandemic).

Finally, the last parameter to be fixed in advance is the mean (*μ*_1_) of the prior distribution for *θ*_1_. This mean was fixed to be *logit(0.1)* to provide a prior estimate of DLT risk on the control arm of 10% based on previous research [28]. Having fixed these parameters, we now calibrate the remaining parameters of the prior distributions of *θ* and the prior estimates of toxicity at each dose.

### Parameter choices based on calibration

The parameters specified in “Parameter choices based on trial setting” are defined by the clinical setting. The rest of the parameters of the design, specifically, the prior distribution hyperparameters (i.e., the parameters of the prior distributions for *θ*) and the prior estimates of toxicity at each dose, define the properties of the design – i.e., how accurate it is and how participants are allocated in the trials (e.g., conservatively or aggressively). There are two approaches for how the dose-toxicity skeleton and hyperparameters can be defined – they can be elicited from clinicians and/or historical data [29], or, if there is no available external information, via calibration [30, 31] to determine the parameters that result in good operating characteristics over a range of plausible scenarios. Alternatively, there might be a “hybrid” approach where the clinicians can provide some constraints that the calibrated prior parameters should satisfy (e.g., the DLT risk on the starting dose is available but not for other doses). For this trial, given the limited reliable knowledge about the safety of Molnupiravir in the new emerging COVID-19 patient population at the time of the trial planning, the calibration approach was taken for both the dose-toxicity skeleton and hyperparameters. The scenarios we used to evaluate performance are given in “Plausible dose-toxicity scenarios”.

We also note that the calibration approach might be selected over elicitation for logistical reasons – while the former can be more computationally expensive, it may be more efficient time-wise than (potentially multiple) discussions with clinical colleagues (particularly relevant in this study). Moreover, there are some recommended design parameter choices [32, 33] for certain dose-escalation models, that again might be a more feasible option with limited funding and/or time. Nevertheless, the model operating characteristics of the chosen parameters should be evaluated under plausible scenarios.

The prior estimates of DLT risk (${p}_j^{(0)}$) at each dose are required to initiate the model and derive the standardised dose levels (note, these are the DLT risks and not the ARDLT). For Molnupiravir, no constraints were placed on the prior DLT risks, other than they were based on a grid of values determined by a prior estimate of DLT risk for the control arm (${p}_0^{(0)}$) and a fixed difference in risk between neighbouring doses, i.e., ${p}_j^{(0)}$ = ${p}_0^{(0)}+\nu \ast j$, where *ν* defines the fixed difference. The values *ν* = {0.05,  0.075, 0.10, 0.125, 0.15} were tried in the grid search. This grid was felt to cover plausible scenarios, where greater differences in DLT risk between doses was thought to be unlikely. The constraint was applied by the statistical team, as equal spacing in the prior DLT risks between the doses reduces the computational complexity of the calibration. In this study, this was deemed appropriate given the actual dosages of the drug were close to each other with no major jumps in dosage. If, however, it is thought a priori that the increase in the toxicity is noticeably higher for one (or more) pair of neighbouring doses, unequal spacing might be needed. However, it is worth noting that the doses should be chosen to cover the part of the dose-toxicity relationship that is of interest (i.e., either side of the targeted toxicity level) with sufficient granularity [34]. Hence, in the case of unequal spacing, it is worth reconsidering the doses so that it is reasonable to consider a priori equal increases in the toxicity risks between neighbouring doses. Finally, also note that this choice of the prior toxicity values does not constrain fixed differences between the estimated DLT risks (or ARDLT) once data is collected.

Next, the joint prior distribution of the model parameters is defined as (*θ*_1_, *log*( *θ*_2_)) ∼ *N*(*μ*, *Σ*) where *μ* = (*μ*_1_, *μ*_2_)^*T*^ is the vector of means, and *Σ* is the covariance matrix with zero covariance and diagonal elements *σ*_1_ and *σ*_2_. Note we use *log*( *θ*_2_) as we require *θ*_2_ ≥ 0 to ensure monotonicity in the risk of toxicity with increasing doses. Assuming 10% toxicity probability in the control arm (as per “Parameter choices based on trial setting”), we set the prior mean for *θ*_1_ to be *μ*_1_ = *logit*(0.1). For the rest of the parameters, the grid of values to be calibrated over were defined as follows:

$${\mu}_2=\left\{-0.15,-0.05,0.00,0.05,0.15\right\}$$

$${\sigma}_1=\left\{0.80,0.90,1.00,1.10,1.20\right\}$$

$${\sigma}_2=\left\{0.10,0.20,0.30,0.40,0.50\right\}$$

**Fig. 1:**
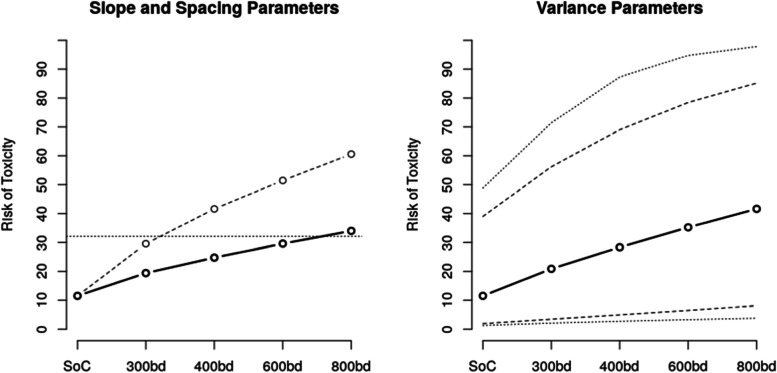
*Left*: Prior dose-toxicity relationship for *σ*_1_ = 1.10, *σ*_2_ = 0.30, with *μ*_2_ =  − 0.15, *ν* = 0.05 (solid line), and *μ*_2_ = 0.15, *ν* = 0.15 (dashed line). The horizontal line is the target toxicity level (note the toxicity on the control arm is not exactly 10% as this is the mean of a nonlinear transformation). *Right*: Bounds of the prior 95% credible interval around prior toxicity estimates for *μ*_2_ =  − 0.05, *ν* = 0.075 with *σ*_1_ = 0.80, *σ*_2_ = 0.10 (dashed lines) and *σ*_1_ = 1.20, *σ*_2_ = 0.50 (dotted lines)

The grids of the values were selected to imply quantitatively different prior shapes of the dose-toxicity relationship (various steepness) and various amounts of uncertainty around the prior estimates (with higher values of variance not necessarily implying greater uncertainty around the prior toxicity estimates [32]). The influence of the prior parameters on the shape of the dose-toxicity relationship and uncertainty around it is illustrated in Fig. 1. These grid values were based on previous experience; in the absence of this, the grid could be determined through an iterative process beginning with a wide range of values and narrowing down to a set that give varying shapes of dose-toxicity relationship and uncertainty in prior estimates.

The left panel of Fig. 1 illustrates two dose-toxicity curves for the values of *μ*_2_ and spacing *ν* on the different edges of the selected grids corresponding to a flat dose-toxicity curve (where the target dose is 800bd) and a steep one (where the target dose is 300bd). The values in-between correspond to the steepness of the dose-toxicity curve between these two cases. The right panel of Fig. 1 illustrates various levels of uncertainty, with the values of hyper-parameters from each extreme of the selected grids.

While the approach taken was based on computational (rather than clinical) considerations, the selected hyperparameters still should imply plausible prior toxicity rates. For example, the ARDLT at the lowest dose should be below the upper toxicity bound *γ*_*toxic*_ = 30% (i.e., assuming the lowest dose will be safe), and the toxicity of the highest dose should not be too low (as this would suggest the study has not been designed appropriately to discover a dose with the desired properties). Note that a low prior probability of toxicity for the highest dose might also result in overly aggressive dose escalation.

Now that a range of parameter values have been defined for the prior DLT risks and hyperparameters, we can evaluate model operating characteristics under a range of plausible scenarios in order to determine the final values of the parameters to be used in the trial.

### Plausible dose-toxicity scenarios

The operating characteristics of the design were extensively evaluated via simulation studies, conducted on a set of clinically plausible dose-toxicity scenarios. As the selection of scenarios can add subjectivity into the assessment of the method’s properties [35] (i.e., one can always find scenarios in which a particular design performs better than others) and one does not know which scenario is true (or closest to the truth), it is important to ensure good operating characteristics over a range of plausible scenarios. To gauge what good operating characteristics are, the non-parametric optimal benchmark [36, 37] can be used. It gives an upper bound of performance and hence large deviation from it suggests poor operating characteristics. The benchmark for binary outcomes is implemented as a web application [38] with the R code for the benchmark with non-binary studies and combination studies freely available online (https://github.com/dose-finding; last accessed 05-Apr-2021). While the benchmark for randomised setting is not yet available, the non-randomised benchmark can still provide a context for the evaluation as it would assess the difficulty of a given scenario. Hence, the binary benchmark [36, 37] for 4 doses targeting an additional toxicity risk of 20% was used. Model performance can also be compared to alternative designs (e.g., an A + B design) to explore if the proposed design is making efficient use of the data within the trial constraints of, e.g., fixed total sample size.

For the current study with 4 dose levels, there are four possible locations of the target dose (provided that at least one dose is safe) – the first, second, third, and fourth dose – and hence four scenarios were considered, where each dose in turn had an ARDLT of 20%. Given the novelty of COVID-19 and of Molnupiravir in these patients, it was assumed that these scenarios were equally likely to be true. Further to the location of the target dose, the DLT rates at non-target doses must be defined (note, these are now what we are assuming to be the truth, and are distinct from the prior estimates we discussed in the previous section).

Generally, when several DLT rates are close to the target value, the proportion of the target dose selection will be lower under a fixed sample size [35]; however, if a range of acceptable DLT risk is defined, the proportion of doses selected that are in this range may still be high if more than one dose falls in this range. Thus, it is accepted that although the “optimal” dose may not be accepted with high probability, a dose with a similar safety (and, assumed, efficacy) profile to this optimal dose has a high chance of being selected. Only larger sample sizes, with its associated logistical downsides, would overcome this issue. As noted previously, DLT rates that are greatly spread out will not provide sufficient granularity for selecting the targeted toxicity level and attempts to avoid this should be made through suitable choice of doses.

The evaluation of model operating characteristics was therefore restricted to scenarios where only one dose was within the target range. Given that the width of the target interval is 10%, a difference of 15% between the target and neighbouring doses was selected (except scenario 1, where the first dose is fixed to be 20% above control). This therefore leads to scenarios where the dose above the target should be deemed too toxic, and doses below the target are implicitly assumed to be too low (i.e., efficacy assumed to be insufficient due to limited DLTs). This resulted in four dose-toxicity scenarios (Table 2) over which the remaining design parameters were calibrated, and which were then used to assess performance of the design (“Model evaluation”). Additionally, Scenario 5 with all doses having toxicity rate above the target toxicity was considered in the evaluations to ensure that the design would stop the trial early if all doses are unsafe. As per “Parameter choices based on trial setting”, the DLT rate on the control arm was 10%.

**Table 2:** Five dose-toxicity scenarios that are considered for the design evaluation. Note, we present the risk of DLT at reach dose here, rather than additional risk of DLT

	*d*_*0*_	*d*_*1*_	*d*_*2*_	*d*_*3*_	*d*_*4*_
Scenario 1	10%	**30%**	45%	60%	70%
Scenario 2	10%	15%	**30%**	45%	60%
Scenario 3	10%	12%	15%	**30%**	45%
Scenario 4	10%	11%	12%	15%	**30%**
Scenario 5	10%	50%	65%	80%	90%

It is worth noting that in the setting with larger numbers of doses or in combination studies, the possible number of target dose locations can be large [36] and it might be infeasible to explore the behaviour of the design over all of them. The recommendation for a comprehensive evaluation would be still to include all clinically plausible scenarios with the main requirement for them to be quantitatively different, e.g., with the target doses being in the difference ranges of the dose grid.

When evaluating the design under different scenarios, various characteristics of the design can be of interest. The primary measure of performance of a design is its accuracy in terms of the proportion of times the correct dose is selected. However, safeguarding of patients is also paramount in Phase I dose-escalation studies; other measures of the performance may be based on safety considerations, such as the average number of DLTs observed in the trial, the average number of patients assigned to toxic doses, and the proportion of overly toxic selection. While all of these characteristics can be informative, we prioritised the proportion of overly toxic selection and overall accuracy. Importantly, the measures of accuracy and safety are conflicting [39], and therefore the final design recommendation will be based on the trade-off between how accurate the selection is against how many patients are exposed to unsafe doses.

### Model evaluation

For a given combination of prior estimates of DLT risk and hyperparameters, the standardised dose levels can be calculated using:

$${d}_j=\frac{logit\ \left\{{\mathrm{p}}_j^{(0)}\right\}-{\mu}_1^{(0)}}{\mu_2^{(0)}},$$

where ${\mu}_1^{(0)}$ and ${\mu}_2^{(0)}$ are prior means of the model parameters.

For each combination of the prior estimates of DLT risk and hyperparameters, the proportion of correct selections (PCS) was computed for each scenario in Table 2. Then, the geometric mean of the PCS across four scenarios was found [31]. The combination of hyperparameters yielding the highest PCS is then selected as the parameters of the operational prior.

Following this procedure, the chosen hyperparameter values were *μ*_2_ =  − 0.05, *σ*_1_ = 1.10, *σ*_2_ = 0.30, and *ν* = 0.075, with the latter implying mean prior DLT risks of 17.5, 25, 32.5, and 40% on doses 1 to 4, respectively (with corresponding ARDLT found by subtracting the control arm mean, 10%). As in other situations where there is a high number of parameters to calibrate, there were several combinations of parameters that led to reasonably similar PCS. Any combination with PCS close to the maximum one can be chosen if it is decided to be more appropriate for the specific setting, though we opted for the maximum here.

**Fig. 2:**
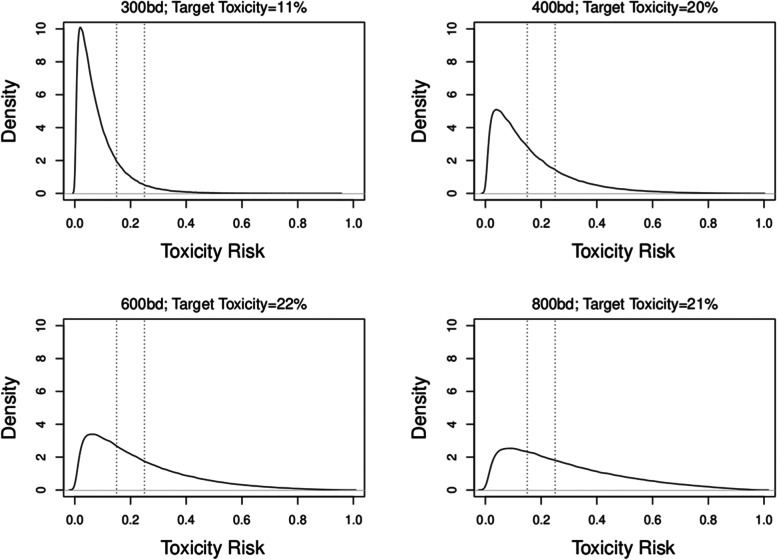
Prior distributions of ARDLT for the 4 doses, based on the calibrated prior parameters. The vertical dotted lines represent the desirable range of additional toxicity (15–25%). The percentages in the header of each graph represent the probability that the ARDLT for the dose lies within this interval

Given the prior distribution of the parameters, *θ*_1_ and *θ*_2_, one can approximate (via sampling) the distributions for the ARDLT at each dose using the dose-toxicity model; this helps to illustrate the implications of the chosen design parameters. The prior distributions on each dose implied by the calibrated prior parameters are given in Fig. 2. This can also be done throughout the trial each time *θ*_1_ and *θ*_2_ are updated.

Figure 2 suggests that the calibrated prior distributions imply a noticeable level of uncertainty around toxicity risks at doses *d*_*2*_*, d*_*3*_*,* and *d*_*4,*_ and less on the lowest dose (that is thought to be safe). The prior probabilities for doses *d*_*2*_*, d*_*3*_*,* and *d*_*4*_ being the target dose were nearly equal to each other (20–22%). The lower probability of the lowest dose being the target will ensure that the escalation is not stuck at the bottom of the grid if it is safe to escalate. Approximately equal prior probabilities of being the target dose is a desirable feature of a calibrated hyper-parameters. If a large discrepancy in these probabilities is observed, a re-calibration for other grid values should be considered.

Plots such as those in Fig. 1 may be useful in communicating DLT risks throughout the trial. However, it is recommended that the trial team undergo some form of training, led by the statistical team, in order to ensure the information conveyed by the plots is clearly understood.

Once the calibrated parameters are found, we can assess model performance against the benchmark (see “Plausible dose-toxicity scenarios”).

### Numerical results

The proportion of each dose selection using the calibrated hyperparameters under all four scenarios are given in Table 3.

**Table 3:** Percentage of correct selections for the calibrated parameters and benchmark results under each scenario

	*d*_*1*_	*d*_*2*_	*d*_*3*_	*d*_*4*_
Scenario 1				
Toxicity Risk	**30%**	45%	60%	70%
Selection Proportion	**59.1%**	32.0%	5.7%	0.0%
Benchmark	**81.0%**	18.8%	0.5%	0.0%
Scenario 2				
Toxicity Risk	15%	**30%**	45%	60%
Selection Proportion	16.9%	**57.4%**	21.4%	3.8%
Benchmark	5.6%	**75.4%**	18.7%	0.2%
Scenario 3				
Toxicity Risk	12%	15%	**30%**	45%
Selection Proportion	2.8%	25.5%	**49.7%**	22.0%
Benchmark	0.2%	4.8%	**76.7%**	18.3%
Scenario 4				
Toxicity Risk	11%	12%	15%	**30%**
Selection Proportion	0.0%	4.8%	28.9%	**65.9%**
Benchmark	0.0%	0.2%	5.1%	**94.4%**

The proportion of optimal dose selection is close to or above 60%, expect for scenario 3. The ratio of PCS to the benchmark is consistent across the scenarios, approximately 65–75%. Given that the standard benchmark does not account for uncertainty in the control arm, this was regarded as acceptable performance and it was concluded that the design is able to identify the correct dose with sufficiently high probability across all considered scenarios. Therefore, we concluded that the design has good properties in terms of the accuracy.

Besides PCS, we also assessed the proportion of overly toxic doses being selected – here it is below 25% under scenarios 2 and 3, which implies that the proportion of overly toxic selection is well controlled under this case. Under the steeply ascending toxicities of scenario 1, the proportion of overly toxic selection is nearly 38%. However, 32% is attributed to the selection of the dose corresponding to 45% of toxicity, or 35% over the control under our assumptions. This is 5% above the maximum acceptable toxicity of *γ*_*toxic*_ = 30%, and therefore harder to distinguish with the limited sample sizes. At the same time, this property of being slightly more aggressive in selections under scenario 1 allows us to limit the proportion of under-toxic selection under flat scenario 3 that ensures that a more promising dose is recommended for the next phase. Lastly, considering the unsafe scenario in which all doses have target toxicity rate above the target one, it was found that the design would terminate the trial with probability 70%; this was agreed to be acceptable in the scope of other operating characteristics and limited sample size.

Considering the results as a whole, and taking into account the context of the design performance and the trade-offs between the accuracy, proportion of over-toxic and under-toxic selections, it was agreed by the statistical and clinical team that the design yields good operating characteristics and can be used to drive dose-escalation decisions in the actual trial.

Importantly, when interpreting the results above, it was assumed that all of the scenarios are equally likely to occur and equally important. It is, however, possible that in some settings some of the scenarios are deemed more probable or clinically important. This can be taken into account when interpreting the result, either formally – for example, considering the weighted mean of the PCS, with the weights reflecting the importance of given scenario – or limiting the proportion of overly toxic selection below some threshold, e.g., 25% – or informally through interpretation of the results under each scenario and discussing the implications with the clinical team.

### Computational aspects

To implement and evaluate the proposed design, R [40] was used. There are existing packages that implement dose-finding model-based designs [41], and the two-parameter logistic model in a dose-finding trial in particular [15, 42]. Most of these are restricted to the non-randomised setting. While these can be modified (through the source code available on CRAN), the Bayesian model was implemented via MCMC algorithm, specifically JAGS [43], via the R package *rjags* [44] without any dose-finding specific packages to enhance flexibility in the implementation. Alternatively, BUGS [45] can be used as it uses similar syntax. The output of the MCMC model is the vectors of the samples of the posterior distribution of the trial parameters, *θ*_1_, *θ*_2_, that are in turn used to compute the samples of the toxicity risk at each dose and control. These posterior distributions are then used for all computations – for example, the probability of being in the target interval is the proportion of posterior samples between 0.15 and 0.25, and the probability of overdosing is the proportion of posterior samples above 0.30.

There are an increasing number of resources (to which this work hopes to contribute) on how model-based dose-finding can be implemented [19]. While the first programming of such designs might be time-consuming for a statistician without prior experience in Bayesian methods, and, specifically, MCMC implementation, we note that it is becoming more common for authors to provide their code for the methods implementation, which may be used a good starting point. We have made our programming available in the form of R code on GitHub at https://github.com/dose-finding/agile-implement. The implementation beyond the first trial/application, however, is associated with diminishing time costs, in our experience.

Another important aspect of implementation is the time it takes to conduct the described calibration and evaluations. As MCMC is used for the implementation, the simulations, with many repetitions, might be quite time consuming. Specifically, the calibration of the proposed design involved 4 parameters with 5 values to be tried. This results in 625 design specifications to be tried for each of the four scenarios. Given the computational complexity and the objective to find the right ballpark of the values implying good operating characteristics, we used 500 simulations and 1000 MCMC samples (with 1000 burn-in) for each combination. These choices for the specified 2-parameter logistic model were found to provide a fair balance between the time taken to evaluate model performance and gaining an adequate understanding of how the PCS changes within each parameter. Paralleling the calibration process (or simply running several R sessions in parallel) resulted in nearly 8 h to complete the calibration across all 4 scenarios (Intel® Core™ i7-8650U CPU @ 1.90GHz × 8).

For the evaluation of the performance of the design under the calibrated operational prior, a larger number of simulations were used to provide more precise estimation on the proportion of each dose selection [46]. Specifically, 2000 simulations, and 5000 MCMC samples were used to provide the results in Table 3. For one scenario, it took around 10 min. On this note, the convergence of the MCMC, in our experience, has not been of a major concern in a dose-finding setting with the two-parameter logistic model.

Finally, for the actual conduct of the trial, 4 ∗ 10^6^ MCMC samples were used to provide estimates of the model for the dose-escalation decision-making. This is chosen to limit the simulation error coming from the Monte Carlo methods.

### Team roles in trial planning

Given the context of the pandemic, speed was of the essence. Therefore, the initial design evaluations, as given in “Application of design to Molnupiravir” onwards, were carried out by the experienced statistical methodologists in the team (PM and TJ). At the time of the planning phase, both PM and TJ were supported by NIHR personal fellowships to provide such statistical support and oversight. The increasing recognition by the NIHR of the resources required to successfully implement such complex designs made the rapid set-up of this trial possible. This also allowed, among other activities, PM to provide training for the AGILE clinical team on Bayesian methods employed in the platform. However, this time might not always be available, and more funding opportunities to allow methodologists and applied statisticians to collaborate in order to support clinical trials in the UK are needed.

At the time of trial planning, the CTU team had limited experience in the methods, amounting to one time-to-event continual reassessment method [47] trial submitted for funding. During study set-up, the methodologists provided a training session to the statisticians in the CTU to support their understanding. This training took the form of a meeting to talk through the broad design and the coding used to produce the results given in “Model evaluation”. The code was passed on to the CTU team (SE and GS) along with some documentation laying out the design and calibration results, allowing them to become familiar with the code and ready to use it during the trial. The methodologists also provided ongoing support during this time, answering queries from the CTU team.

### Timescales

The general AGILE platform design (as mentioned in “Parameter choices based on trial setting”) was carried out prior to the final protocol for Molnupiravir, where the clinical and practical considerations were decided on. Otherwise, given the context of the trial, the calibrations were undertaken largely in parallel with development of the protocol, and the protocol outlined the intended philosophy of the analysis and when the model would be updated rather than details of the model. A separate document was produced once calibration was completed and final model parameters were determined. In other circumstances, this evaluation would occur prior to the protocol being finalised and should be included in the protocol; indeed, some funding bodies will ask for these evaluations to be completed at the application stage.

## Results

### Trial processes

The trial design for Molnupiravir involved cohorts of six participants, randomised 2:1 to Molnupiravir or standard of care. The Safety Review Committee (SRC) were to review data following 7 days of follow-up of the last participant in each cohort (dosing was carried out over 5–6 days). At this stage, the Bayesian dose-toxicity model was updated based on observed DLTs (defined according to grade 3 events using CTCAE v5.0). The model was used to support decision-making to either escalate, de-escalate, recruit another cohort at the same dose, stop the study due to unacceptable toxicity, or recommend a dose for testing in Phase II. Note, the SRC did not have to follow the model recommendations and could choose the dose they felt most appropriate. Deviating from the model’s recommendation alters the operating characteristics (e.g., the PCS may be altered), but the model can, however, continue to be used after a diverging decision is taken by the SRC as the model can accommodate such deviations.

### Team roles in trial delivery

The CTU team were responsible for delivery of the trial, running the analysis in preparation for the SRC meetings and presenting results. GS and SE took responsibility for this within the CTU, investing time in understanding the code, carrying out trial runs, and linking this to data pulled from the trial database. The code was automated to return formatted output in order to produce results very rapidly (within hours) following the end of data collection. PM developed additional code in order to run a second, independent set of analyses as a check for the analysis run by the CTU team, also implemented in *rjags*. This was not linked directly to the database and was run based on the CTU providing the minimum amount of information (numbers per arm and number of DLTs per arm).

The methodologists attended the SRCs, with TJ, as a member of the SRC, providing additional input in presenting and interpreting the results. PM provided an additional training session during the study to support the understanding of the model outputs for the clinical members of the SRC. Additional time was given in the first SRC meeting for SE and TJ to describe the model and its results in order to support the understanding of voting members; brief reminders were given in subsequent meetings.

### SRC report template

A template SRC report was developed prior to recruitment, led by the CTU with input from the methodologists (and the SRC). The part of the SRC report concerning the model-based recommendation is provided in Additional file 1. The report included estimates of the DLT rate for each dose (including standard of care), ARDLT for each dose of Molnupiravir, and the probability of ARDLT being 30% or more. The estimates of DLT risks were based on the mean of the posterior distribution for each dose and were presented alongside 95% credible intervals. The relationship between doses and risk of DLT was presented in a plot (i.e., a line plot of a section of the two-parameter logistic model), alongside 95% credible intervals (with a reference line for 20% ARDLT over standard of care). The model was used to derive the next recommended dose, determined by the dose with highest probability of lying within 15–25% of ARDLT over standard of care. The SRC report included this recommendation, along with the probability the ARDLT was within this range for the recommended dose.

### SRC reports in practice

The model outputs were presented for two situations: 1) based on all available information on DLTs at the time of the analysis; and 2) based on the first 7 days of follow-up only. The latter was used to provide a potentially fairer comparison of arms, as it was likely that follow-up across groups would be of notably different lengths (as the control arm included participants across cohorts, and so would include participants with the full 28 days of follow-up) and hence estimates of ARDLT may be confounded by time. The plots for each dose are given in the main trial publication [48] to demonstrate how the model updated over time. Note that throughout the study, no DLTs were observed.

In one cohort, one participant received only a subset of the full dosing schedule (two of ten intended doses) for reasons other than DLTs. They were, by the definitions given in the protocol, evaluable and so were counted as one of six in the cohort to be reviewed by the SRC. However, it was not immediately obvious if this person should be included in the model. As they did not experience a DLT before stopping treatment, they should in theory be included as not having a DLT – but as a result, the treatment may appear to be safer than it was (i.e., the model does not acknowledge the fact this person received only two doses). For this situation, the SRC was presented with three different scenarios:

1. where the participant was included as a person not experiencing a DLT;
2. where the participant was included as a person experiencing a DLT; and
3. where the person was not included in the model.

This approach may be considered a sensitivity analysis and allowed the SRC to understand the impact on DLT estimates (and potentially their decision-making) depending on how this participant was treated in the modelling. In this instance, how this participant was included made little difference to the overall results and the decision of the SRC (see Additional file 2 for the results under each scenario above).

Following escalation to, and evaluation of one cohort at, the highest dose, and with no observed DLTs, the SRC had two realistic options: 1) to enrol an additional cohort to the highest dose, or 2) close Phase I and recommend this highest dose for testing in Phase II. The model recommended the highest dose be tested next (note, the model is not designed to recommend ending Phase I). As well as having the highest probability of being in the 15–25% ARDLT range, the model suggested the highest dose had a very low probability of having ARDLT of 30% or more over control, suggesting this dose was likely to be safe.

To support the results at this point, the SRC were also presented with what-if scenarios, where different hypothetical results of an additional cohort of six participants were fed into the model – ranging from zero DLTs to everyone on treatment experiencing a DLT (see Additional file 2). Estimates of DLTs, credible intervals, probability of being too toxic (ARDLT of 30% or more), and recommended doses were presented under each scenario. The model provided reassurance that at this point in the trial, only an extreme result (all four people in the treatment arm experiencing a DLT and none in the control arm) would result in the model recommending a different dose (i.e., to de-escalate one dose level). In the opinion of the SRC, this information, coupled with the perceived unlikeliness of this outcome, provided reassurance that the existing data was sufficient to recommend the highest dose for Phase II testing. It is worth noting that this additional evaluation was carried out in the SRC meeting itself, given that the code had been well prepared in the run up to the trial. This approach has parallels with the dose transition pathways advocated elsewhere [49], allowing the research team to understand progression of the trial under hypothetical examples.

### Statistical analysis plan

The statistical analysis plan (SAP) was developed during the trial; as with the SRC template, this was led by the CTU statisticians with input from the methodologists and clinical team. The section of the SAP relating to the dose-escalation model is given in Additional file 1. There are currently limited guidelines on what should be reported from model-based dose escalation trials, so we hope this provides a useful guide for future trials.

### Changes to an ongoing trial

One challenge faced in the trial was occasional changes to some of the design parameters after calibration was completed. Early in trial development, the number of doses (and their magnitude) was not settled, due to accruing information from other ongoing trials (e.g., healthy volunteer trials). The code to calibrate the design parameters was, however, developed early and could be quickly adapted to changes in the number and magnitude of the doses. These changes could be quickly incorporated while the study was underway, even after recruitment had begun. Generally, if there are minor changes to the number of dose levels and re-calibration cannot be done due to time/resource constraints, one can still utilise previously calibrated parameters. While the performance might not be optimal, the model-based design would still be expected to have good operating characteristics. Nevertheless, a simulation study under the new number of doses (and specified parameters) is still essential to conduct to ensure good statistical properties of the design. If an analysis has been carried out (e.g., for an SRC), we would strongly discourage re-calibrating the model as this may involve ad hoc changes to the prior information that may be influenced in light of observing data.

Other changes to an ongoing trial that can be straight-forwardly accommodated include the cohort size and the maximum sample size. Again, while the performance of the model-based design might not be optimised under the changed circumstances, the operating characteristics of the design are not expected to deviate noticeably from that previously found. More generally, the decision making of the SRC cannot always be captured a priori by the statistical team and so the calibrations are conducted under fixed behaviour that may not perfectly mimic practice. Although this may impact the operating characteristics somewhat, the model can still be used to derive estimates of toxicity.

New doses can be added during a trial, though careful consideration would need to be given to determining the standardised dose levels for the newly introduced doses. The existing structure may be used to define the new dose levels (e.g., preserving the spacing when the new dose levels are outside the existing range, or using linear interpolation within the existing range proportional to the actual dosage). However, the similarity (or otherwise) of the new dose(s) to existing doses can affect how the model performs, and so testing and evaluation of the model across a wider range of scenarios (e.g., with the DLT risk for the new dose equal to the target toxicity level) is required to ensure satisfactory performance.

Changes in other design parameters after the first analysis has already taken place, however, might have undesirable effect, e.g. the target toxicity rate, overdose control, upper toxicity bound and tolerance around the target rate as these, essentially, correspond to change of the objective of the trial and, hence, should be avoided where reasonably could be. If agreed to be essential, changes in these will require the recalibration of the parameters (possibly considering the currently observed data) to align the specification of the design with the amended objectives and further evaluations before the decision on further escalation is made.

## Discussion

We have described the use of a model-based dose-escalation model delivered as a collaboration between experienced adaptive trials methodologists and UKCRC Clinical Trials Unit (CTU) statisticians. We have highlighted many of the practical considerations encountered through the study that may serve to support others attempting to implement these designs, as well as demonstrating the benefits of such models.

Model-based dose-escalation designs have been shown to bring benefits over traditional A + B designs; however, as may be obvious from the Methods section, there are many (and sometimes complex) aspects to consider that may serve to limit their use. We have laid out a template (Table 1 and accompanying discussion) to further guide users in approaching such designs. We hope this guide will help potential users understand who in the research team needs to input, and when, to the parameter choices, in order to allow an efficient and effective approach to design. We also hope that the discussion of the scenarios in which to calibrate and evaluate the model will guide potential users to prepare models most capable of achieving their aims. This paper also provides guidance on how to present the ongoing results of the trial to the Safety Review Committee, as well as what information may be needed in the SAP/final trial report (see Additional file 1); note, the published results are also given in the final trial publication [48].

We appreciate the circumstances of having expert methodological input may be rare in many circumstances. From the perspective of the CTU statistical team, this expert input brought two primary benefits: 1) the speed at which the trial could be designed – in terms of the methodologists having existing code, knowing how to efficiently approach the process of eliciting prior information and other considerations, and how best to approach the calibration (e.g., what scenarios to test); and 2) a “safety net” for any questions or uncertainties that arose throughout the trial (which motivated this paper). In more usual circumstances (i.e., not in a pandemic), we believe it is possible for less experienced trial teams to design these trials, but it should be acknowledged that the first attempt will require an investment of time by a statistical team. However, this investment will, in our experience, diminish each time these designs are used. Even given this, we encourage the research community to recognise the complexity of these trials, and believe that infrastructure, such as that provided by the NIHR fellowships for TJ and PM, will be crucial in making use of these designed more widespread and ensuring they maximise their potential.

Lastly, and importantly, we would like to emphasise the success of the model in this trial in terms of how it was received by the whole trial team and its ability to impact decision-making. The clinical team become increasingly confident in the model, both in terms of it matching their intuitions (hence increasing their trust) and in terms of being able to interpret the results. The what-if scenarios presented in “SRC reports in practice” were very important following dosing at the highest dose level; this allowed the team to consider the possible outcomes from recruiting another cohort, and the associated pros and cons (e.g., additional information from this cohort versus the additional resource required to recruit). Given the model had aligned with their intuitions to that point, they could put faith in the outcomes of these what-if scenarios, and were able to see a quantification of toxicity estimates and their uncertainty rather than relying on potentially different interpretations of what, for example, a DLT in the treatment arm might mean for their decision making.

## Conclusion

Model-based designs for dose-finding studies have important theoretical and practical advantages over rule-based methods, and we encourage those designing dose-finding studies to invest time in learning these methods. We hope that this paper serves to address many of the questions that may arise when using such designs – both during the design and implementation – so that we can encourage others to consider using these methods, to improve the accuracy when determining suitable doses for future efficacy testing.

## Supplementary Information

**Additional file 1.** Safety Review Committee Report Template and Statistical Analysis Plan.

**Additional file 2.** What-if scenarios

## Data Availability

Code is provided on GitHub: https://github.com/dose-finding/agile-implement
