# Supplementary material for: Practical recommendations for implementing a Bayesian adaptive phase I design during a pandemic
Source: BMC Med Res Methodol. 2022 Jan 20;22:25. doi: 10.1186/s12874-022-01512-0 (PMC8771176; doi:10.1186/s12874-022-01512-0)
Supplement: Supplementary file 1 — Additional file 1. Safety Review Committee Report Template and Statistical Analysis Plan. [file 12874_2022_1512_MOESM1_ESM.docx]

Appendix A – Safety Review Committee Report Template and Statistical Analysis Plan

The content below is for all accumulated data at the time (for the final analysis, this would be all data); the same content was repeated for 7 days from initiation of treatment for the SRC reports. The evaluable population was all those randomised to the control arm plus anyone randomised to, and receiving one dose of, treatment.

*SRC report text*

Using the DLT data from all accumulated data from first dose, the estimated risk of toxicity for the current dose is xx%, and the estimated additional toxicity (i.e., toxicity beyond that of the control arm) is xx%. The probability that the additional toxicity for the current dose level is 30% or more is xx%. The probability that the additional toxicity for the current dose lies in the interval 15-25% is xx%. A summary of the estimated toxicity and additional toxicity rates based on all the accumulated data are given in Table X and the estimated dose-toxicity model is given in Figure X.

According to the model, the next recommended dose level is xx mg. This dose has estimated additional toxicity (i.e., toxicity beyond that of the control arm) of xx%. The probability that the additional toxicity for this recommended dose is 30% or more is xx%. The probability that the additional toxicity for this recommended dose is in the interval 15-25% is xx%.

*SRC and SAP tables and figures*

*Table X: Estimated toxicity and additional toxicity rates for the control arm and tested EIDD-2801 doses using all accumulated data.*

| **Dose level** | **Estimated DLT rate**  **(95% HPD credible interval)** | **Estimated additional toxicity above control (%)** | **Probability of additional toxicity ≥30% over control arm** |
| --- | --- | --- | --- |
| Control | xx.x%  (xx.x – xx.x) | --- | --- |
| Molnupiravir 300mg | xx.x%  (xx.x – xx.x) | xx.x% | xx.x% |
| Molnupiravir 400mg | xx.x%  (xx.x – xx.x) | xx.x% | xx.x% |
| Molnupiravir 600mg | xx.x%  (xx.x – xx.x) | xx.x% | xx.x% |
| Molnupiravir 800mg | xx.x%  (xx.x – xx.x) | xx.x% | xx.x% |


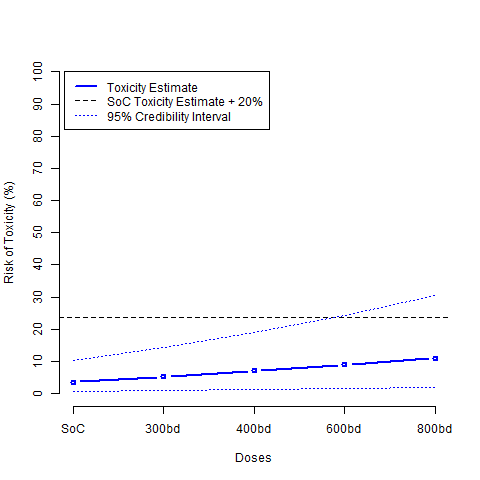


**[EXAMPLE: dose-toxicity graph]**

*Figure X: Dose-toxicity curve based on all accumulated data*
