# Supplementary material for: Practical recommendations for implementing a Bayesian adaptive phase I design during a pandemic
Source: BMC Med Res Methodol. 2022 Jan 20;22:25. doi: 10.1186/s12874-022-01512-0 (PMC8771176; doi:10.1186/s12874-022-01512-0)
Supplement: Supplementary file 2 — Additional file 2. What-if scenarios [file 12874_2022_1512_MOESM2_ESM.docx]

Appendix B – What-if scenarios

The Bayesian dose-toxicity has been run several times based on a number of “what if” scenarios, assuming additional data has been accrued based on each of the below scenarios (Table 1). The results from each scenario are summarised in Table 2.

*Table 1: Scenarios*

| 1 | Including person who withdrew from treatment (i.e., 4 people assessed at 800mg BD^1^) |
| --- | --- |
| 2 | Excluding person who withdrew from treatment (i.e., 3 people assessed at 800mg BD) |
| 3 | Treating person who withdrew from treatment as a DLT (i.e., 4 people assessed at 800mg BD, with one DLT) |
| 4 | Additional cohort of 6 people at 800mg BD^2^ with no DLTs |
| 5 | Additional cohort of 6 people at 800mg BD^2^ with 2 people having at least one DLT in the treatment arm |
| 6 | Additional cohort of 6 people at 800mg BD^2^ with 4 people having at least one DLT in the treatment arm |

^1^BD = twice daily. ^2^Assumes 6 people randomised in 2:1 ratio to EIDD 800mg BD or standard of care, under scenario 1.

*Table 2: Results*

| Scenario | Mean toxicity | Upper 95% credible interval | Target dose probability (15-25%  additional toxicity) | Probability of additional toxicity ≥30% over control arm (%) |
| --- | --- | --- | --- | --- |
| 1 | 11.0 | 30.5 | 8.6 | 0.9 |
| 2 | 11.8 | 32.9 | 10.0 | 1.4 |
| 3 | 17.4 | 42.3 | 20.8 | 4.8 |
| 4 | 8.7 | 23.5 | 4.3 | 0.2 |
| 5 | 18.6 | 40.5 | 25.4 | 4.6 |
| 6^1^ | 31.5 | 57.7 | 34.0 | 29.3 |

^1^In this scenario, the recommended dose would be 600mg BD. In all other cases, 800mg would be recommended. (Note: the model is not designed to explicitly recommend progression to phase II – only the next dose.)
